# Bi-directional regulation functions of lanthanum-substituted layered double hydroxide nanohybrid scaffolds *via* activating osteogenesis and inhibiting osteoclastogenesis for osteoporotic bone regeneration

**DOI:** 10.7150/thno.56607

**Published:** 2021-05-03

**Authors:** Min Chu, Zhenyu Sun, Zhanghao Fan, Degang Yu, Yuanqing Mao, Yaping Guo

**Affiliations:** 1 The Education Ministry Key Lab of Resource Chemistry and Shanghai Key Laboratory of Rare Earth Functional Materials, Shanghai Normal University, Shanghai 200234, China.; 2 Shanghai Key Laboratory of Orthopedic Implants, Department of Orthopedic Surgery, Shanghai Ninth People's Hospital, Shanghai JiaoTong University School of Medicine, Shanghai 200011, China.

**Keywords:** lanthanum, bone regeneration, osteoclastogenesis, osteogenesis, osteoporosis

## Abstract

**Rationale:** Osteoporotic patients suffer symptoms of excessive osteoclastogenesis and impaired osteogenesis, resulting in a great challenge to treat osteoporosis-related bone defects. Based on the positive effect of rare earth elements on bone metabolism and bone regeneration, we try to prove the hypothesis that the La^3+^ dopants in lanthanum-substituted MgAl layered double hydroxide (La-LDH) nanohybrid scaffolds simultaneously activate osteogenesis and inhibit osteoclastogenesis.

**Methods:** A freeze-drying technology was employed to construct La-LDH nanohybrid scaffolds. The *in vitro* osteogenic and anti-osteoclastogenic activities of La-LDH nanohybrid scaffolds were evaluated by using ovariectomized rat bone marrow stromal cells (rBMSCs-OVX) and bone marrow-derived macrophages (BMMs) as cell models. The *in vivo* bone regeneration ability of the scaffolds was investigated by using critical-size calvarial bone defect model of OVX rats.

**Results:** La-LDH nanohybrid scaffolds exhibited three-dimensional macroporous structure, and La-LDH nanoplates arranged perpendicularly on chitosan organic matrix. The La^3+^ dopants in the scaffolds promote proliferation and osteogenic differentiation of rBMSCs-OVX by activating Wnt/β-catenin pathway, leading to high expression of ALP, Runx-2, COL-1 and OCN genes. Moreover, La-LDH scaffolds significantly suppressed RANKL-induced osteoclastogenesis by inhibiting NF-κB signaling pathway. As compared with the scaffolds without La^3+^ dopants, La-LDH scaffolds provided more favourable microenvironment to induce new bone in-growth along macroporous channels.

**Conclusion:** La-LDH nanohybrid scaffolds possessed the bi-directional regulation functions on osteogenesis and osteoclastogenesis for osteoporotic bone regeneration. The modification of La^3+^ dopants in bone scaffolds provides a novel strategy for osteoporosis-related bone defect healing.

## Introduction

Osteoporosis that is one of the most prevalent skeletal disorders occurs commonly in postmenopausal women, elderly people and the patients with nutritional imbalance or deficiency 1-3. Under osteoporotic conditions, the osteogenic differentiation ability of bone marrow stromal cells (BMSCs) is seriously hindered, while osteoclast-related bone resorption is excessively activated. The disruption of bone remolding equilibrium results in the progressive loss of bone mass and microstructural degeneration 4. Osteoporotic patients significantly raise the risk of bone defects 5. Although various osteoclast inhibitors and osteogenesis promotors such as bisphosphonates, calcitonin, parathyroid hormone and estrogen are employed to prevent osteoporosis 6,7, they are not efficient for the treatment of osteoporotic bone defects. Therefore, it is urgent to design the bone scaffolds that simultaneously activate osteogenesis and inhibit osteoclastogenesis.

Rare earth elements (REEs) that accumulate in human body within the lifespan, participate in body metabolism, anti-inflammation, sterilization, anti-tumor, immunoregulation and tissue regeneration 8-10. Lanthanum (La), a member of REEs family, is used as a bioactive element for bone disease treatment and bone defect repair 11-13. Lanthanum carbonate, serving as a calcium-free phosphate binder, effectively reduces serum phosphate levels in the patients with chronic kidney disease (CKD), delays the development of vascular calcifications, and prevents the progression of CDK-mineral and bone disorder 11,14. Lanthanum chloride (LaCl3) attenuates osteoclastogenesis and bone resorption via inhibiting nuclear factor-κB (NF-κB) signaling pathway, and suppresses the titanium particle-induced bone loss 12. The osteogenic differentiation and extracellular matrix mineralization of BMSCs are remarkably enhanced by La^3+^ ions 13. According to the positive effect of La element on regulating osteoblast-osteoclast balance, it is reasonally inferred that La-doped bone scaffolds are fit to treat osteoporotic bone defects even under the pathological states of impaired osteogenesis and excessive osteoclastogenesis.

Traditional bone repair materials such as β-tricalcium phosphate and hydroxyapatite, showing similar chemical elements to bone minerals, exhibit desirable cytocompatibility and osteoconductivity 15-17. However, they lack sufficient abilities to activate osteogenesis and inhibit osteoclastogenesis for osteoporotic bone regeneration 15-17. Upon to now, layered double hydroxide (LDH, [M_1-*x*_^2+^N*_x_*^3+^(OH)_2_]*^x^*^+^[A*_x/n_^n-^*]*^x-^·m*H_2_O) have been employed for bone repair application 18-20. Divalent M^2+^ and trivalent N^3+^ cations are substituted by various metal ions such as Mg^2+^, Cu^2+^, Fe^2+^, Al^3+^, La^3+^ and Fe^3+^, while A*^n^*^-^ interlayer anions represents Cl^-^, CO_3_^2-^, SiO_3_^2-^ and NO_3_^-^ 21. Thanks to the tunable chemical compositions, LDHs are endowed with unique biological characteristics via the introduction of bioactive metal ions 22,23. Mg^2+^ ions play a vital role in balancing osteoblast/osteoclast differentiation. Mg deficiency in bone tissues easily causes the disorder of bone remodeling and increases osteoporosis risk 24. MgAl-LDHs up-regulates the expression of osteogenesis-related genes including Runx-2, OSX and OCN by activating c-Jun N-terminal kinase (JNK) and extracellular signal-regulated kinases (ERK) signaling ways 25. The biological effects of La^3+^ ions depend on the concentrations, namely, low concentrations promote cell proliferation while high concentrations exert cell apoptosis 26. The modification of La^3+^ ions in MgAl-LDHs may effectively prevent the rapid release, and thus avoids the toxic side effects.

**Scheme 1:**
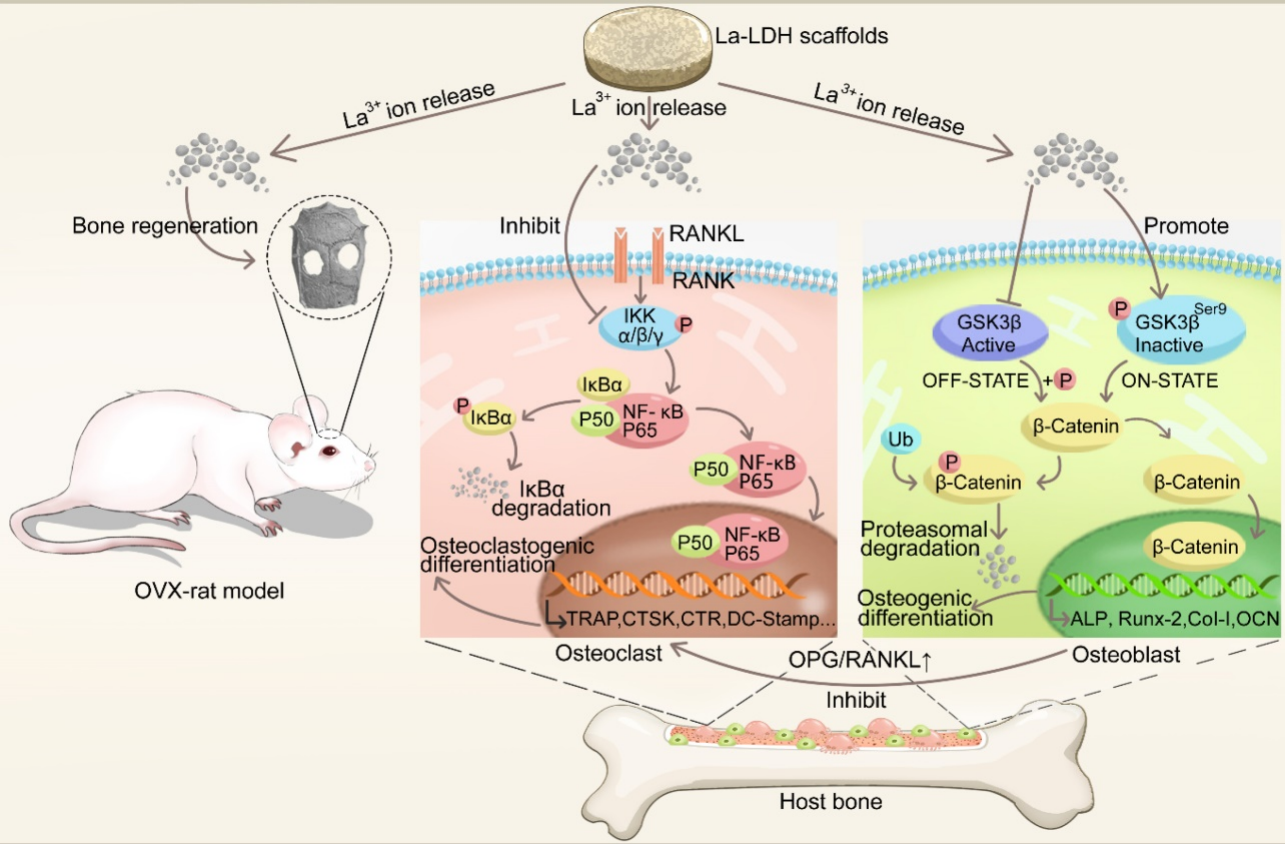
Illustration of the biological effects of La-LDH scaffolds. The functional scaffolds promote osteogenic differentiation of rBMSCs-OVX by activating Wnt/β-catenin pathway, and suppress RANKL-induced osteoclastogenesis by inhibiting NF‐κB signaling pathway.

Chitosan (CS), one of natural cationic polysaccharides, is similar to structural characteristics of glycosaminoglycans in extracellular matrix (ECM) 27,28. Herein, a freeze-drying technology was used to fabricate La-doped LDH (La-LDH) nanohybrid scaffolds by using CS as organic matrix. Previous works have reported that La^3+^ dopants can regulate host-to-scaffold immune responses, recruit endogenous stem cells, enhance phagocytic activity of macrophages, promote osteogenesis and angiogenesis for bone regeneration 29-32. In this work, we hypothesized that La-LDH nanohybrid scaffolds possessed bi-directional regulation functions on osteogenesis and osteoclastogenesis (Scheme 1). To prove the hypothesis, the regulation mechanism of La-LDH scaffolds on osteoblasts and osteoclasts was investigated by using ovariectomized rat BMSCs (rBMSCs-OVX) and bone marrow-derived macrophages (BMMs) as cell models. Interestingly, the La-LDH scaffolds not only promoted the osteogenic differentiation of rBMSCs-OVX via activating Wnt/β-catenin pathway, but also suppressed RANKL-induced osteoclastogenesis via inhibiting NF-κB pathway. Moreover, the osteoporotic bone regeneration ability of La-LDH nanohybrid scaffolds was evaluated by a critical-sized OVX rat calvarial defect model.

## Methods

### Synthesis of La-LDH nanoplates

LDH and La-LDH nanoplates were prepared by coprecipitation method. For the La-LDH, part of Al^3+^ ions in LDH were substituted by La^3+^ ions and Mg^2+^ ions did not have any change. The La-LDH with the Al/La molar ratios of 7:1 and 4:1 was abbreviated as La1/7-LDH and La1/4-LDH, respectively. The Mg/(Al+La) molar ratio was controlled at 3:1 for all groups. Briefly, Mg(NO_3_)_2_·6H_2_O, Al(NO_3_)_3_·9H_2_O and La(NO_3_)_3_·6H_2_O were dissolved in 300 mL of deionized water for ultrasound to form a salt solution (Table S1). 9.60 g NaOH was dissolved in 150 mL deionized water. The salt solution and alkali solution were simultaneously dropped into a three-necked flask with 150 mL of deionized water. The chemical reaction was carried out at 40 °C for 3 h and then at 65 °C for 24 h. The pH value of the mixed solution was kept at 10.0~11.0. Finally, the LDH and La-LDH products were washed with deionized water and dried at 60 °C for 48 h.

### Preparation of La-LDH nanohybrid scaffolds

LDH, La1/7-LDH and La1/4-LDH nanohybrid scaffolds were prepared by a freeze-drying technology. 2.0 g CS was completely dissolved in acetic acid solution (50 mL, 2.0 vol%) under mechanical agitation. 2.0 g LDH, La1/7-LDH and La1/4-LDH nanoplates were added into the CS solution, respectively. The mixture was mechanically stirred for 2.5 h, followed by the injection in a 24-well plate. The samples were dried at -60 °C in a freeze-drying equipment for 48 h, soaked in 5 wt% NaOH for 24 h, and washed with deionized water up to pH = 7.0. Finally, the LDH, La1/7-LDH and La1/4-LDH nanohybrid scaffolds were freeze-dried again.

### Material characterization

The phases of LDH, La1/7-LDH and La1/4-LDH scaffolds were analysed by an X-ray diffraction (XRD, D/MAX-111 C, Japan) with Cu Kα radiation (λ = 1.542 Å) operating at 40 kV and 40 mA. The morphological images of La-LDH scaffolds were acquired by scanning electron microscopy (SEM; JSM-6380LV, Japan), and their chemical elements were detected by energy-dispersive spectrometry (EDS). Transmission electron microscopy (TEM; JEOL2100, Japan) with selected area electron diffraction (SAED) was employed to analyse the microstructure of the La-LDH nanoplates in La-LDH scaffolds. Thermogravimetry analysis (TG-DTA, PerkinElmer instrument) of La1/4-LDH scaffolds was detected under an air flow of 50 mL/min from 25 °C to 800 °C with a heating rate of 10 °C/min. The compressive strengths of LDH, La1/7-LDH and La1/4-LDH scaffolds (*R* = 0.75 cm, *h* = 1.5 cm) were detected by microcomputer control electronic universal testing machine (WDW-0.5C, Shanghai Hualong Microelectronics Co. Ltd., China) with a compression speed of 5 mm/min. *In vitro* ion release performances of LDH, La1/7-LDH and La1/4-LDH scaffolds were tested by soaking 0.4 g samples in 9 mL ultrapure water at 37 °C. The concentrations of Mg^2+^ and La^3+^ ions were analysed by inductively coupled plasma/optical emission spectrometry (ICP/OES; Perkin Elmer, OPTIMA 3300 DV).

### Cell culture

All cell and animal experiments were approved by the Animal Research Committee of Shanghai Ninth People's Hospital affiliated to Shanghai Jiao Tong University, School of Medicine. To create osteoporotic rat models, 16-week-old female Sprague-Dawley (SD) rats were given a bilateral ovariectomy (OVX) by two dorsal incisions 33. BMSCs were isolated from the hind limbs of OVX rats according to the previous report 34. Briefly, bilateral femurs and tibias were collected under aseptic conditions. The bone marrow was flushed out with 10 mL Dulbecco's modified Eagle's medium (DMEM, Gibco, USA) supplemented with 10% fetal bovine serum (FBS, Gibco, USA) and antibiotics (penicillin 100 U/mL, streptomycin 100 U/mL). The cells were cultured in a 5% CO_2_ incubator at 37 °C, and non-adherent cells were discarded after 48 h. The culture medium was renewed every 2 days. As the cells reached a confluence of 80% to 90%, they were passaged in a 0.25% trypsin/EDTA medium. The rBMSCs-OVX of passages 2-3 were employed for *in vitro* cell tests without adding any other osteogenic supplement. Primary BMMs were extracted from long bone marrow of 6-week-old C57/BL6 male mice by flushing out bone marrow cells from femurs and tibiae 35. Isolated BMMs were incubated in α-MEM supplemented with 10% FBS, 1% penicillin/streptomycin and 30 ng/mL M-CSF. The cell cultures were maintained in a humidified incubator with 5% CO_2_ at 37 °C. The LDH, La1/7-LDH or La1/4-LDH scaffolds with the diameter of 5 mm and thickness of 2 mm were used for the following cell tests.

### *In vitro* cytocompatibility evaluation

The cell viability of rBMSCs-OVX and BMMs was assayed by Cell Counting Kit-8 (CCK-8; Dojindo Molecular Technologies, Inc., Japan). Briefly, 1.0×10^4^ rBMSCs-OVX were cultured with LDH, La1/7-LDH or La1/4-LDH scaffolds in a 24-well plate for 1, 4 and 7 days, respectively. 1.0×10^4^ BMMs were cultured with LDH, La1/7-LDH or La1/4-LDH scaffolds for 2, 3 and 4 days, respectively. The above culture medium was replaced every 2 days. At different time points, the rBMSCs-OVX or BMMs on the scaffolds were washed by phosphate buffered saline (PBS). The cell culture media containing 10% CCK-8 solutions were given to the cells. After incubation for 2 h at 37 °C, the optical density was measured at 450 nm (630 nm reference) by a microplate reader (*n* = 6).

The LDH, La1/7-LDH or La1/4-LDH scaffolds were placed in a 24-well plate, followed by seeding 2.0×10^4^ rBMSCs-OVX. After cultured for 3 days, the samples were fixed with 2.5% glutaraldehyde for 20 min, rinsed twice with PBS, dehydrated with graded concentrations of ethanol solution, and freeze-dried at -80 °C. The rBMSCs-OVX on the scaffolds were observed by SEM (JSM-6380LV, Japan).

### *In vitro* osteogenic differentiation experiments

5×10^4^ rBMSCs-OVX and bone scaffolds were placed in the lower chamber and the upper chamber of a 24-well transwell plate, respectively. The culture medium was renewed every 2 days. After 7 days, the rBMSCs-OVX were fixed with 4% paraformaldehyde. Alkaline phosphatase (ALP) staining procedure was performed according to the manufacturer's instructions (Shanghai Hongqiao Medical Reagent Company, Shanghai, China). Moreover, the ALP activity of rBMSCs-OVX was quantitatively determined. The cells were rinsed three times with PBS and lysed in 0.2 % TritonX-100. The samples were equivalently mixed with p-nitrophenyl phosphate (pNPP, Beyotime Biotechnology, China) and incubated for 60 min at 37 °C. The optical density of ALP protein was determined at 405 nm. Total protein content was measured by BCA protein assay kit (Beyotime Biotechnology, China). ALP activity was calculated by normalizing to the total protein contents.

rBMSCs-OVX were cultured with LDH, La1/7-LDH and La1/4-LDH scaffolds in a 24-well transwell plate for 21 days, respectively. The culture medium was renewed every 2 days. The rBMSCs-OVX were fixed with 4% paraformaldehyde for 20 min, stained with 1 mM alizarin red solution (Cyagen, USA) for 10 min and rinsed with PBS three times. The alizarin red staining images were acquired by an inverted bright field microscope (Leica, Germany).

To measure the expression levels of osteogenesis-related genes, 2×10^5^ rBMSCs-OVX were cultured with LDH, La1/7-LDH and La1/4-LDH scaffolds for 7 days, respectively. The culture medium was replaced every other day. Total mRNA was extracted by TRIzol Reagent (Invitrogen, USA). Complementary DNA (cDNA) was synthesized by using 1.0 mg RNA and Prime-Script RT Master Mix (Takara). Quantitative real-time polymerase chain reaction (qRT-PCR) analysis was carried out to detect the gene expression of ALP, runt-related transcription factor 2 (Runx-2), osteocalcin (OCN), collagen type I (COL-I), osteoprotegerin (OPG) and RANKL by an ABI 7500 Sequencing Detection System. GAPDH served as the housekeeping gene for normalization. The primer sequences used for rBMSCs-OVX were listed in Table S2.

Western blot assay was performed to quantify the protein expression of rBMSCs-OVX. 2×10^5^ rBMSCs-OVX were cultured with LDH, La1/7-LDH or La1/4-LDH scaffolds in 6-well plates for 14 days. The culture medium was replaced every other day. Total proteins in rBMSCs-OVX were extracted by RIPA cell lysis buffer (Beyotime, China) containing protease inhibitor cocktail and phosphatase inhibitor cocktail, followed by centrifugation at 4 °C for 15 min. The supernatants were collected, and protein concentrations were determined by BCA Protein Assay Kit (Beyotime Biotechnology, China). 20 μg protein extracts were loaded on 10% SDS-PAGE gel and then electro-transferred to a 0.22 µm PVDF membrane. After blocking in 5% non-fat milk in Tris-buffered saline with 0.1% tween (TBST) for 2 h, the membranes were incubated with primary antibody against GSK-3β, phosphorylated GSK-3β (p-GSK-3β), β-catenin, Runx-2, OPG, RANKL and β-actin (Cell Signaling Technology, Shanghai, China) at 4 °C overnight. The membranes were visualized with secondary antibody conjugated to horseradish peroxidase (Jackson ImmunoResearch Laboratories, Inc., USA) using ECL plus reagents (Solarbio, China) under a Tanon 5200 imaging system. Protein expression levels were normalized to β-actin.

### *In vitro* RANKL-induced osteoclastogenesis assays

Osteoclast differentiation of BMMs was visualized by tartrate-resistant acid phosphatase (TRAP) staining. 5.0×10^4^ cells/well BMMs were cultured with LDH, La1/7-LDH and La1/4-LDH scaffolds in a 24-well transwell plate for 24 h, respectively. The BMMs were supplied with α-MEM containing 30 ng/mL M-CSF and 50 ng/mL RANKL. The culture medium containing M-CSF and RANKL was renewed every other day. TRAP staining was conducted by an acid phosphatase kit (Sigma, USA) at day 7. The TRAP-positive multinucleated cells that contained more than 3 nuclei were counted as osteoclasts. These osteoclasts were photographed by an optical light microscope (Olympus, Tokyo, Japan). The total area of TRAP-positive regions and the total number of osteoclasts were quantified by an Image J software (NIH, Bethesda, MD).

3.0×10^5^ cells/well BMMs were cultured with the LDH, La1/7-LDH and La1/4-LDH scaffolds in the presence of M-CSF (30 ng/mL) and RANKL (50 ng/mL) until mature osteoclasts were formed at day 7. Total RNA was extracted by TRIzol reagent (Invitrogen, Life Technologies, USA). cDNA was synthesized. The expression of osteoclastogenesis-associated genes including NFATc1, c-fos, Cathepsin K (CTSK), calcitonin receptor (CTR), TRAP, V-ATPase d2 and DC-STAMP was evaluated by quantitative real-time PCR assay. The primer sequences used for mouse osteoclasts were shown in Table S3. The relative expression of each target gene was normalized to GAPDH.

To investigate the effects of La-LDH scaffolds on RANKL*‐*induced signaling, total cellular proteins (TCPs) were extracted from different time points. 3.0×10^5^ cells/well BMMs were pre-treated with extracts of the LDH, La1/7-LDH and La1/4-LDH scaffolds for 4 h, followed by stimulation with 50 ng/mL RANKL for 0, 5, 10, 20, 30 and 60 min (short time course). The BMMs at a density of 3.0×10^5^ cells/well were stimulated with the extracts of La1/4-LDH or LDH scaffolds in the presence of RANKL for 0, 1, 3, and 5 days (long time course). Proteins were collected from the cells. After electrophoresis and transmembrane, the PVDF membranes were incubated with specific primary antibodies against NF-κB-p65, p-NF-κB-p65, IκBα, p-IκBα, c-fos, NFATc1 and β-actin. The membranes were probed with appropriate secondary antibody, followed by the detection of antibody reactivity and visualization of protein bands by Odyssey V3.0 image scanning (Li-Cor Inc., Lincoln, USA).

### *In vivo* reconstruction of calvarial defects of OVX rats

After general anesthesia with pentobarbital sodium (40 mg/kg), OVX rat calvarial defects with two full-thickness defects of 5 mm diameter were created by an electric trephine drill. Thirty-six critical-sized calvarial defects in eighteen OVX rats were randomly filled with LDH, La1/7-LDH and La1/4-LDH scaffolds, respectively. After the scaffolds were implanted in defect regions, the periosteum was repositioned and the incision was closed sutured by 4-0 silk sutures. Each animal was received an intraperitoneal injection of antibiotics. At 14 days and 7 days before sacrifice, the rats were injected intraperitoneally injections with 10 mg/kg calcein and 30 mg/kg alizarin red, respectively. At 12 weeks of post-implantation, all the rats were sacrificed by the overdose injection of pentobarbital. The cranium tissues were harvested and fixed in 4% paraformaldehyde solution.

### Micro-CT reconstruction analysis

The cranium tissues were examined by micro-CT (μCT-100, SCANCO, Switzerland) with a resolution of 18 μm at an X-ray voltage of 70 kV and a current of 200 μA. In order to quantitatively analyze newly mineralized bone tissues, the bone mineral density (BMD) and the ratio of bone volume to tissue volume (BV/TV) were analyzed by a CTAn software.

### Histological analysis

Calvarial specimens were dehydrated in ethanol and then embedded in polymethylmethacrylate. Three coronal sections with thicknesses of 150 µm were obtained by a diamond saw (SP1600, Leica), followed by grinding and polishing to a final thickness of ~40 µm. The fluorescent labeling images were obtained by a confocal laser scanning microscope (CLSM, Leica, Germany). The average distances between two fluorochromes were calculated by BIOQUANT OSTEO 2019 software. The mineralization rates (μm/day) were calculated by dividing the average distances by 14 days. The sections subjected to Von Kossa staining were detected by an optical microscope (Leica, Germany). The newly formed bone area was quantified by Image Pro Plus 6.0 (Media Cybernetic, USA). For histology analysis, formalin-fixed samples were decalcified in 10% EDTA solution for 4 weeks, followed by embedding in paraffin. Sections with a thickness of 4 μm were stained with Masson's trichrome. For immunohistochemical assay, paraffin sections were deparaffinated, antigens were retrieved, and subsequently the sections were incubated with 5% bovine serum albumin (BSA) for 10 min to block nonspecific staining. The slices were incubated with primary antibodies against Col-1 and OCN (Abcam) overnight at 4 °C. The sections were incubated with biotinylated secondary antibody and stained with 3,3'-diaminobenzidine (DAB) substrate. The stained images were detected by an optical microscope (Leica, Germany).

### Statistical analysis

All data were presented as means ± standard deviation (SD) from at least 5 independent experiments. Statistical analyses were performed in SPSS 20.0 software using one-way analysis of variance (ANOVA) or unpaired Student's t-test. The value of *p* < 0.05 was considered as a statistical significance.

## Results

### Morphology and structure of La-LDH nanohybrid scaffolds

**Figure 1:**
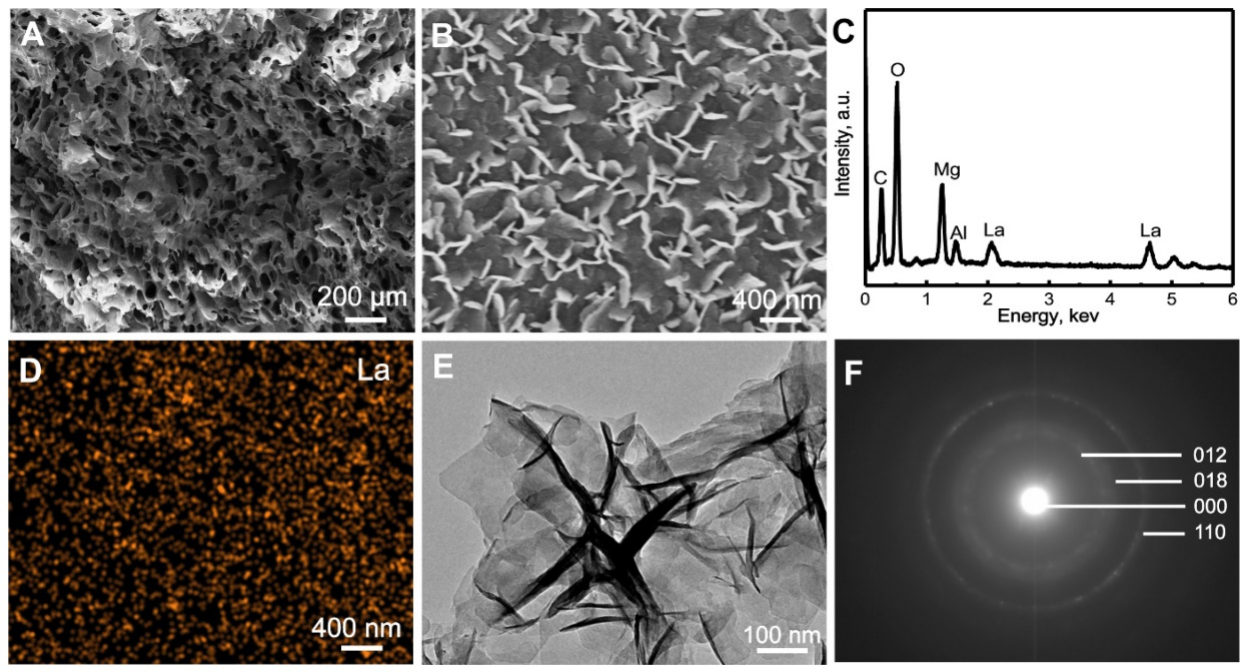
Characterization of La1/4-LDH scaffolds: (A, B) SEM images, (C) EDS pattern, (D) La distribution map, (E) TEM images and (F) ED pattern.

The La1/7-LDH and 1/4La-LDH nanohybrid scaffolds were constructed by the two procedures: (i) the coprecipitation preparation of La-LDH nanoplates; and (ii) the freeze-drying fabrication of La-LDH scaffolds using La-LDH and CS as raw materials. During the freeze-drying process, the sublimation of ice crystals led to forming interconnected macropores with size of 100~200 μm (Figures 1A and S1). The La-LDH nanoplates arranged vertically on the surfaces of CS matrix (Figures 1B and and S1D). The ED pattern indicated that the La1/4-LDH nanohybrid scaffolds consisted of La, C, O, Mg and Al elements. The C element was ascribed to CS, while the La, Mg and Al elements were ascribed to La-LDH nanoplates (Figure 1C). The La element was distributed throughout the scaffolds, as confirmed by the La distribution map (Figure 1D). The TEM image revealed that the La1/4-LDHs in the scaffolds possessed two-dimensional plate-like shape, which agreed with the SEM images (Figure 1B and E). The ED pattern indicated the visible diffraction rings of LDHs, which were ascribed to (012), (018) and (110) crystal planes (Figure 1F). All the LDH, La1/7-LDH and La1/4-LDH nanohybrid scaffolds showed the similar morphologies, suggesting that La^3+^ dopants had no obvious effects on the macroporous structure and LDH plate-like shape (Figures 1 and S1).

**Figure 2:**
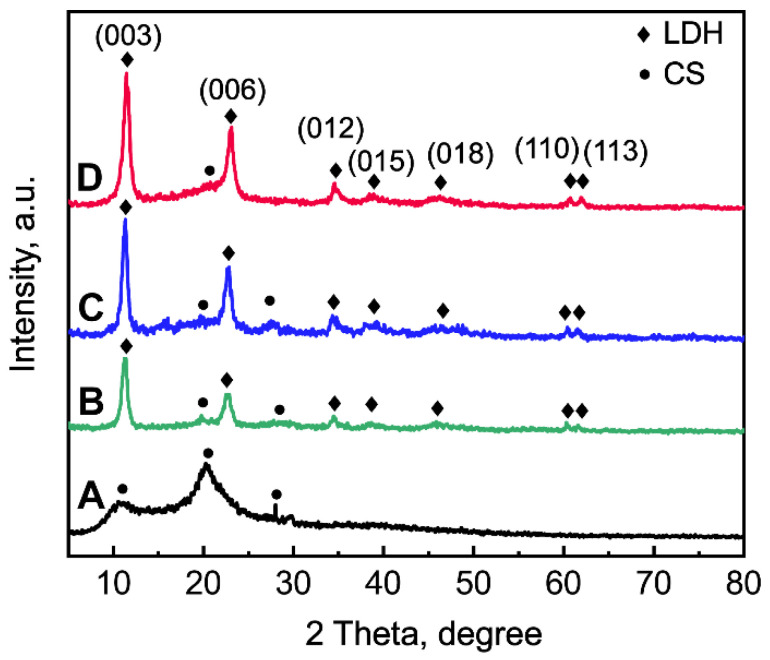
XRD patterns of samples: (A) CS powders, (B) LDH scaffolds, (C) La1/7-LDH scaffolds, (D) La1/4-LDH scaffolds.

The phases of La1/7-LDH and La1/4-LDH nanohybrid scaffolds were characterized by XRD patterns using CS powders and LDH scaffolds as control groups (Figure 2). The CS was a semi-crystalline material, whose XRD characteristic peaks located at 10.9°, 20.3° and 28.1° (Figure 2A). After the combination of CS with La-LDH (or LDH) nanoplates in the scaffolds, the characteristic peaks of both materials were detected in the XRD patterns (Figure 2B and C). For the LDH scaffolds, the peaks at 2*θ* = 11.4°, 23.0° and 34.9° corresponded to (003), (006) and (012) planes of LDHs, suggesting the layered hydrotalcite structure. The basal spacing (*d*_003_) of LDHs was calculated as 0.776 nm, which was determined by the thickness of NO_3_^-^ anion layer and metal hydroxide layer 36. The characteristic peaks of La1/7-LDH and La1/4-LDH scaffolds were much similar to those of LDH scaffolds (Figure 2B and C). Notably, the basal spacing (*d*_003_) of the La1/4-LDH in the scaffolds was 0.783 nm, which was slightly greater than that of LDHs because of the partial substitution of Al^3+^ ions by La^3+^ ions.

### Thermal behaviour, compression strength and* in vitro* degradability of La-LDH nanohybrid scaffolds

**Figure 3:**
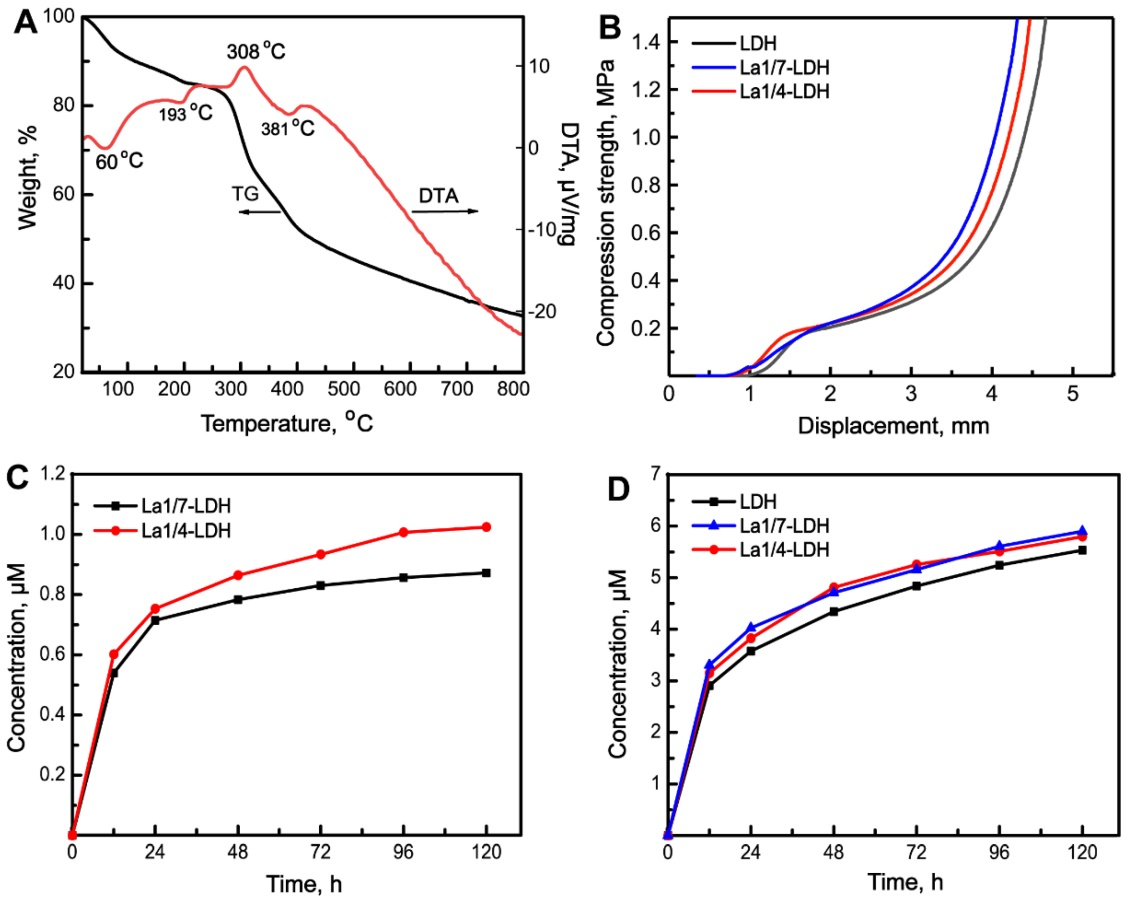
**Thermal behaviour, compression strength and* in vitro* degradability of La-LDH nanohybrid scaffolds.** (A) TG-DTA images of La1/4-LDH scaffolds; (B) the compression curves of LDH, La1/7-LDH and La1/4-LDH scaffolds; (C) the release curves of La^3+^ ions from La1/7-LDH and La1/4-LDH scaffolds; (D) the release curves of Mg^2+^ ions from the LDH, La1/7-LDH and La1/4-LDH scaffolds.

The thermal behaviour of La1/4-LDH nanohybrid scaffolds was analyzed by TG-DTA curves (Figure 3A). Lots of water molecules were physically adsorbed by the La1/4-LDH scaffolds due to the presence of -NH_2_ and -OH functional groups, and interlayer water existed within the La1/4-LDH nanoplates. The weight loss of 18.0 wt% in the range of 25~250 °C was ascribed to the evaporation of adsorbed water and interlayer water, whose endothermic peaks in the DTA curve located at around 60 °C and 193 °C, respectively (Figure 3A). The weight loss of 52.0 wt% was detected in the range of 250~800 °C. On the one hand, the thermal decomposition of CS in the scaffolds occurred over 250 °C, as confirmed by the exothermic peak at around 308 °C. On the other hand, the weight loss of the La-LDHs in the scaffolds took place due to the dehydroxylation and the thermal decomposition of interlayer NO_3_^-^ anions 37. The corresponding endothermic peak was detected at around 381 °C. The final thermal decomposition products of the La1/4-LDH scaffolds were only metal cation oxides including MgO, La_2_O_3_ and Al_2_O_3_.

Inspired by the spongy structure of cancellous bones with porosities of 50~90%, artificial bone scaffolds should possess three-dimensional macropores supporting nutrient transport and bone tissue in-growth 38. The pure CS porous scaffolds showed the ductile feature, easily leading to the failure of macropore structure 16,39. Interestingly, the incorporation of LDH, La1/7-LDH or La1/4-LDH nanoplates within the CS scaffolds improved the mechanical property of organic/inorganic scaffolds (Figure 3B). As the external forces were exerted to the nanohybrid scaffolds, the macropores were firstly damaged. The compression strengths of all the LDH, La1/7-LDH and La1/4-LDH scaffolds were approximately 0.21 MPa (Figure 3B), which could meet the clinic requirement of bone repair for non-load-bearing bones 40.

Artificial bone scaffolds must have appropriate biodegradation rates that match well with the growth rates of new bone tissues 41. The* in vitro* degradability of LDH, La1/7-LDH and La1/4-LDH nanohybrid scaffolds were evaluated by soaking them in a release medium, and the concentrations of La^3+^ and Mg^2+^ ions were detected by ICP at the as-given time points (Figure 3C and D). The ion release profiles indicated that both the La^3+^ and Mg^2+^ ions showed the high release rates in the first 48 h. As the dissolution-reprecipitation process of the La1/7-LDH and La1/4-LDH nanoplates in the scaffolds arrived gradually at an equilibrium state, the release rates of La^3+^ and Mg^2+^ ions began to decrease (Figure 3C and D). For the La-LDH nanoplates, the Al^3+^ ions in the LDH crystals were partly substituted by La^3+^ ions. Because the La^3+^ doping amounts in the La1/4-LDH scaffolds were greater than those in the La1/7-LDH scaffolds, the release concentrations of La^3+^ ions from the former were higher than those from the latter (Figure 3C). All the LDH, La1/7-LDH and La1/4-LDH scaffolds have the same doping amounts of Mg^2+^ ions, so they showed the similar release performances (Figure 3D). Notably, the concentrations of La^3+^ and Mg^2+^ ions from the La1/4-LDH scaffolds reached 0.35 μM and 5.90 μM at the condition of the ions release equilibrium, respectively. The fact that the equilibrium concentrations of Mg^2+^ ions were remarkably greater than the La^3+^ ions was due to the following reasons: (i) the original amounts of Mg^2+^ ions were greater than the La^3+^ ions for the preparation of La-LDHs; and (ii) trivalent La^3+^ ions showed the stronger electrostatic binding with the -OH groups in LDHs than the divalent Mg^2+^ ions, leading to the lower release rates.

### Cytocompatibility of La-LDH nanohybrid scaffolds

**Figure 4:**
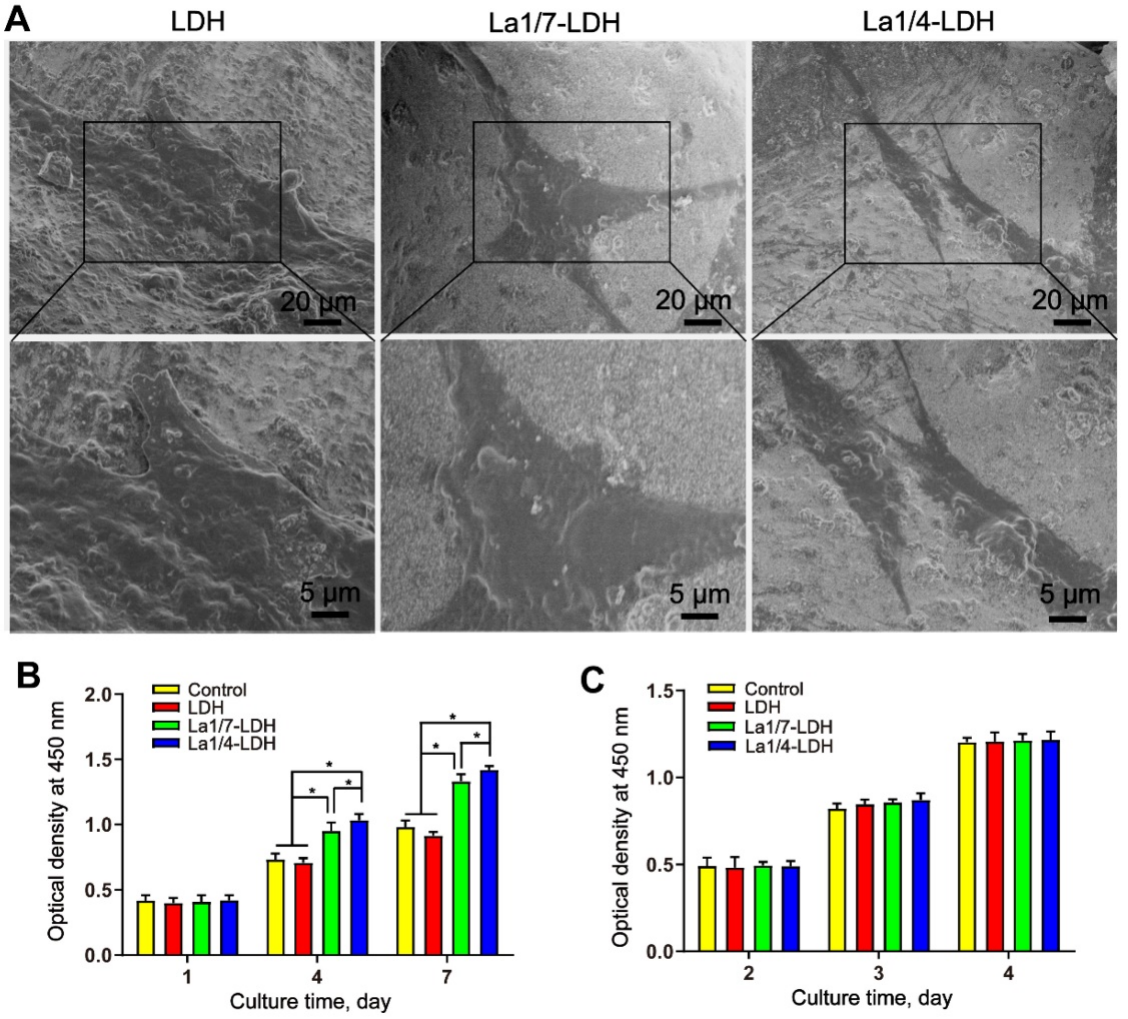
**La-LDH nanohybrid scaffolds supported cell adhesion and proliferation.** (A) SEM images of rBMSCs-OVX cultured with bone scaffolds for 3 days. The proliferation of (B) rBMSCs-OVX and (C) BMMs cultured with blank control, LDH, La1/7-LDH and La1/4-LDH nanohybrid scaffolds (*p < 0.05,* n* = 6).

BMMs and rBMSCs-OVX were employed as cell models to investigate the cytocompatibility of LDH, La1/7-LDH and La1/4-LDH nanohybrid scaffolds. After cultured for 3 days, the rBMSCs-OVX with a flattened shape adhered firmly to the scaffold surfaces (Figure 4A). The rBMSCs-OVX on the LDH, La1/7-LDH and La1/4-LDH scaffolds exhibited pseudopodia-like structures. The CCK-8 assays revealed that all the scaffolds facilitated the proliferation of rBMSCs-OVX, and the cell number gradually increased as the culture time was prolonged from 1 day to 7 days (Figure 4B). Interestingly, the La^3+^ dopants in the La-LDH scaffolds possessed the positive effects on the rBMSCs-OVX proliferation. The cell number on the La-LDH group was significantly greater than that on the blank control and LDH groups especially at the time points of 4 days and 7 days (*p < 0.05), and it increased with the doping amounts of La^3+^ ions. Moreover, the LDH, La1/7-LDH and La1/4-LDH nanohybrid scaffolds had non-cytotoxicity to BMMs, as confirmed by the increased cell number with time (Figure 4C). However, no significant difference of BMMs number was detected among the blank control, LDH, La1/7-LDH and La-LDH groups (Figure 4C), suggesting that the La^3+^ dopants did not affect the proliferation of BMMs.

### La-LDH nanohybrid scaffolds promoted osteogenic differentiation of rBMSCs-OVX

**Figure 5:**
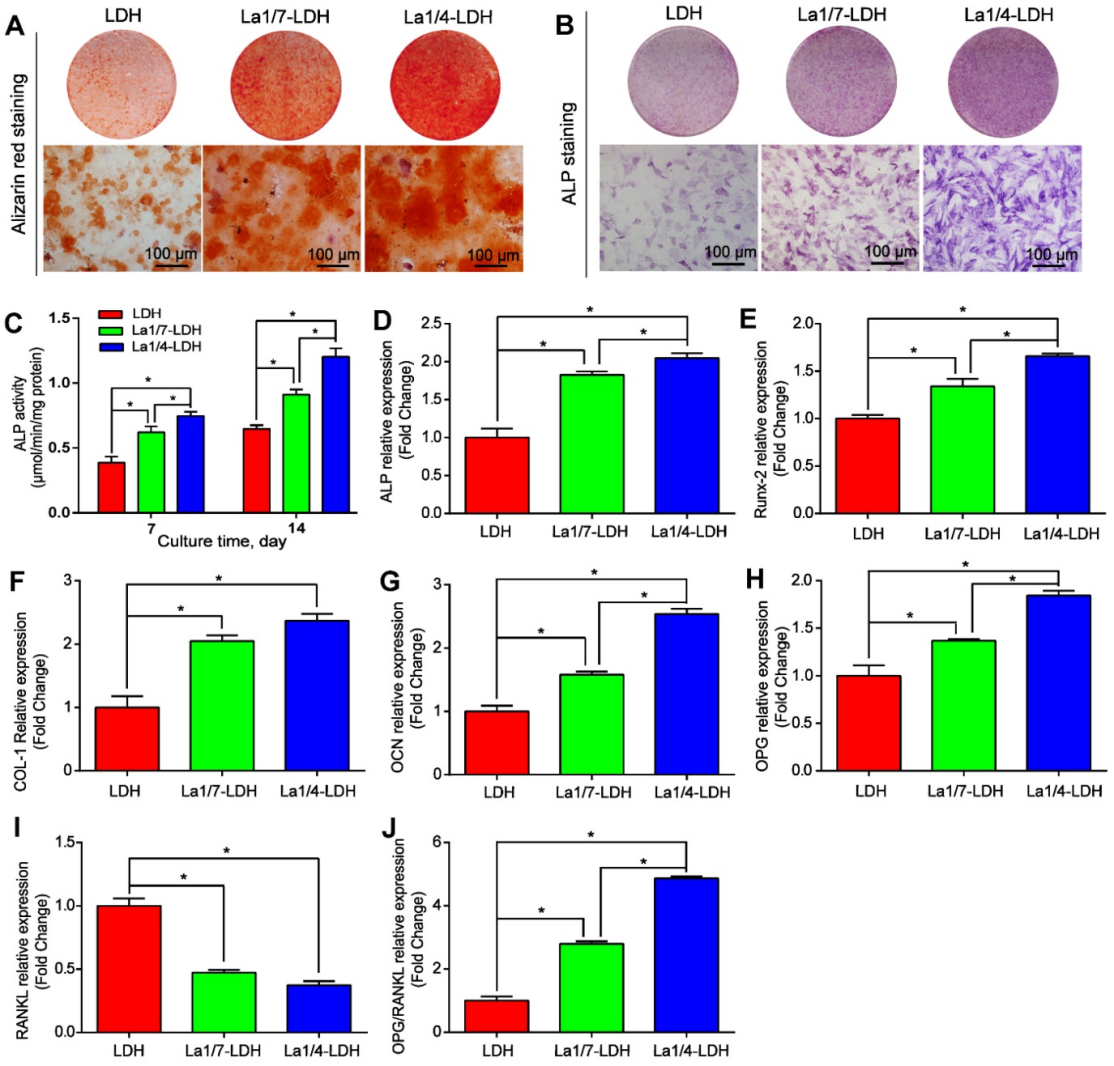
**La-LDH nanohybrid scaffolds promoted osteogenic differentiation of rBMSCs-OVX.** (A) Alizarin red staining images and (B) ALP staining images of the rBMSCs-OVX as-treated with La-LDH and LDH scaffolds at days 21 and 7, respectively. (C) ALP activity of rBMSCs-OVX as-treated with different scaffolds for 7 and 14 days (*p < 0.05). The qRT-PCR results for the mRNA expression of osteogenic-related genes in rBMSCs-OVX: (D) ALP, (E) Runx-2, (F) COL-I, (G) OCN, (H) OPG, (I) RANKL and (J) OPG/RANKL after 7 days of incubation time (*p < 0.05).

The osteoinductivity of La-LDH nanohybrid scaffolds were characterized according to the osteogenic differentiation ability of rBMSCs-OVX (Figure 5). The deposition amounts of calcium nodules in ECM were employed as a marker of osteogenesis 40. The alizarin red staining images suggested that more calcium nodules were detected in the La1/7-LDH and La1/4-LDH groups than those in the LDH group, and the ECM mineralization had a positive correlation to La^3+^ doping amounts (Figure 5A). The ALP that could effectively regulate phosphate metabolism was regarded as an early marker to evaluate osteogenic differentiation. As compared with the LDH scaffolds, the La^3+^ dopants in the La-LDH nanohybrid scaffolds made the ALP images become more intensive (Figure 5B). The ALP quantitative results further confirmed that the ALP activity of rBMSCs-OVX cultured with the LDH and La-LDH scaffolds increased with prolonging the culture time from 7 days to 14 days. Moreover, the ALP activity enhanced with the increase of La^3+^ amounts in bone scaffolds (Figure 5C). These results demonstrated that the La^3+^ dopants in the La-LDH nanohybrid scaffolds contributed to ALP activity and ECM mineralization (Figure 5A-C).

The expression levels of ALP, Runx-2, OCN, COL-I, OPG and RANKL genes were characterized by qRT-PCR after rBMSCs-OVX were cocultured with different scaffolds for 7 days (Figure 5D-J). The ALP, Runx-2, OCN, COL-I and OCN genes were the pivotal markers of osteogenic differentiation, whose expression levels were remarkably up-regulated with the increase of La^3+^ amounts (*p* < 0.05, Figure 5D-H). The osteogenic capacity of the bone scaffolds was as followed: La1/4-LDH scaffolds > La1/7-LDH scaffolds > LDH scaffolds. RANKL, a type II transmembrane protein, played a key role in inducing osteoclast differentiation and maturation 42. As compared with the LDH scaffolds, the La-LDH nanohybrid scaffolds remarkably down-regulated the expression levels of RNKL (*p* < 0.05, Figure 5I), suggesting that the La^3+^ dopants possessed a great potential in inhibiting bone loss and osteoporosis occurrence. OPG, a secreted decoy receptor of RANKL, contributed to regulating bone resorption 43. OPG expression levels and OPG/RANKL ratios increased with La^3+^ dopants in a dose-dependent manner, demonstrating that the La-LDH nanohybrid scaffolds had better anti-osteoclastogenic ability than the LDH scaffolds (Figure 5H and J).

**Figure 6:**
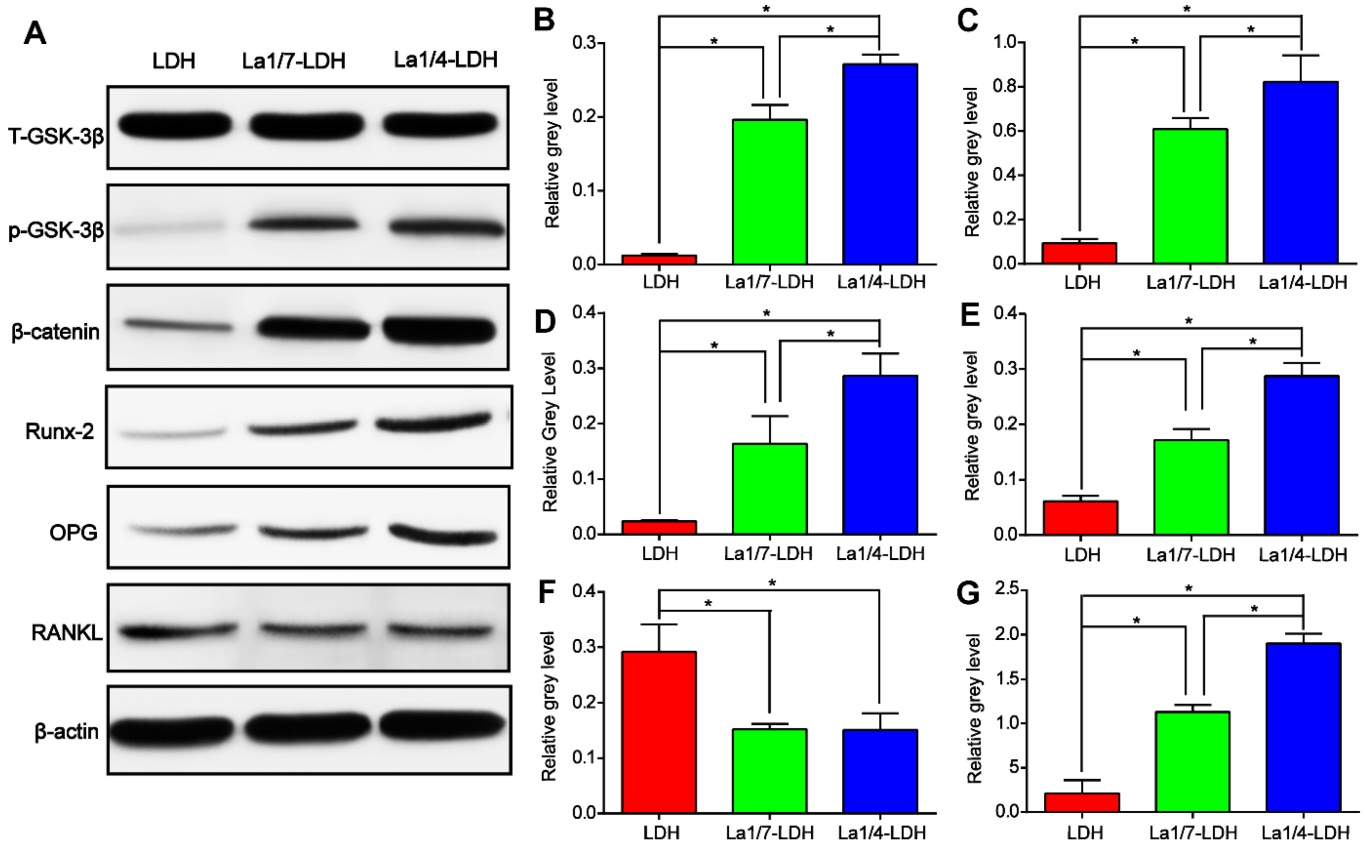
**La-LDH nanohybrid scaffolds activated Wnt/β-catenin signaling pathway and up-regulated OPG/RANKL.** (A) Western blot results for t-GSK-3β, p-GSK-3β, β-catenin, Runx-2, OPG and RANKL expression of the rBMSCs-OVX cultured with La-LDH and LDH scaffolds for 14 days. β-actin was used as the internal reference. (B) The level of p-GSK-3β was normalized to t-GSK-3β (*p < 0.05). The levels of (C) β-catenin, (D) Runx-2, (E) OPG, (F) RANKL and (G) OPG/RANKL were normalized to β-actin (*p < 0.05).

Western blot assay was performed in order to reveal the effect mechanism of La^3+^ dopants on the enhanced osteogenic capacity of the La-LDH nanohybrid scaffolds (Figure 6). As compared with LDH scaffolds, the La-LDH scaffolds remarkably increased the p-GSK-3β kinase level of rBMSCs-OVX, bringing about the accumulation of β-catenin which initiated the transcription of osteogenesis-related genes (Figure 6A). These findings were confirmed by the paralleled increase in the protein levels of Runx-2 and OPG as well as the decrease in RANKL level in both the La1/4-LDH or La1/7-LDH groups (Figure 6A and F). Moreover, La^3+^ dopants in the La-LDH nanohybrid scaffolds influenced the expression levels of the above proteins in a dose-dependent manner, as determined by quantitative analysis results (Figure 6B-G). According to the PCR and Western blot results, it could be inferred that the stimulatory effect of La-LDH nanohybrid scaffolds on osteogenic differentiation of rBMSCs-OVX was associated with the activation of Wnt/β-catenin signaling pathway and the up-regulation of OPG/RANKL.

### La-LDH nanohybrid scaffolds inhibited RANKL-mediated osteoclastogenesis

**Figure 7:**
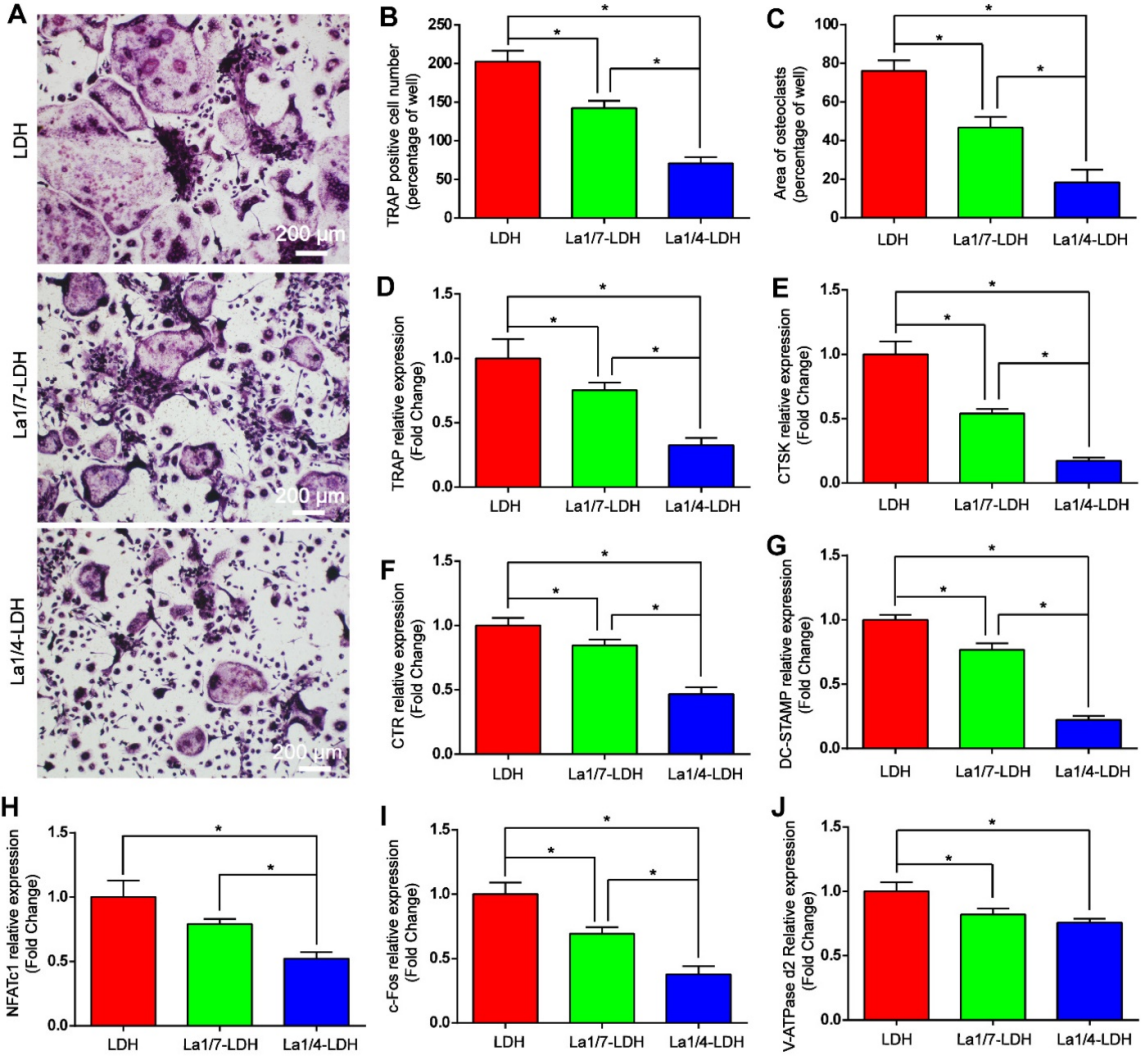
**La-LDH nanohybrid scaffolds showed anti-osteoclastogenic effects.** (A) TRAP staining images of BMMs cultured with La-LDH and LDH scaffolds in the presence of M-CSF (30 ng/mL) and RANKL (50 ng/mL) for 7 days. (B) Numbers and (C) areas of TRAP‐positive osteoclasts (*p < 0.05). The qRT-PCR results of expression of osteoclast marker genes including (D) TRAP, (E) CTSK, (F) CTR, (G) DC-STAMP, (H) NFATc1, (I) c-Fos and (J) V-ATPase d2 (*p < 0.05).

The effects of La-LDH nanohybrid scaffolds on osteoclast differentiation were systematically evaluated* in vitro* by using BMMs as cell models (Figure 7). TRAP image indicated that abundant typical pancake‐shaped multinucleated osteoclasts were formed on the surfaces of the LDH scaffolds after 7 days (Figure 7A). In contrast, the number and area of TRAP‐positive multinucleated osteoclasts remarkably decreased with the increase of La^3+^ dopants in the scaffolds (Figure 7B and C). The La1/7-LDH scaffolds mildly suppressed osteoclast formation with approximately 30% reduction in the number of mature osteoclasts. Notably, the La1/4-LDH nanohybrid scaffolds almost completely inhibited osteoclast formation, and almost no round osteoclast was observed in the TRAP image (Figure 7A). The inhibitory effect of La^3+^ dopants on osteoclastogenesis were further investigated by qRT-PCR assay (Figure 7D-J). As compared with the LDH scaffolds, the La-LDH scaffolds significantly down-regulated the expression levels of NFATc1, c-fos, CTSK, CTR, TRAP, V-ATPase d2 and DC‐STAMP. Among three groups, La1/4-LDH nanohybrid scaffolds achieved the strongest inhibitory effect on osteoclastogenesis. Collectively, the La-LDH nanohybrid scaffolds exhibited anti-osteoclastogenic effects in a dose‐dependent manner via inhibiting RANKL‐induced osteoclast formation and growth.

**Figure 8:**
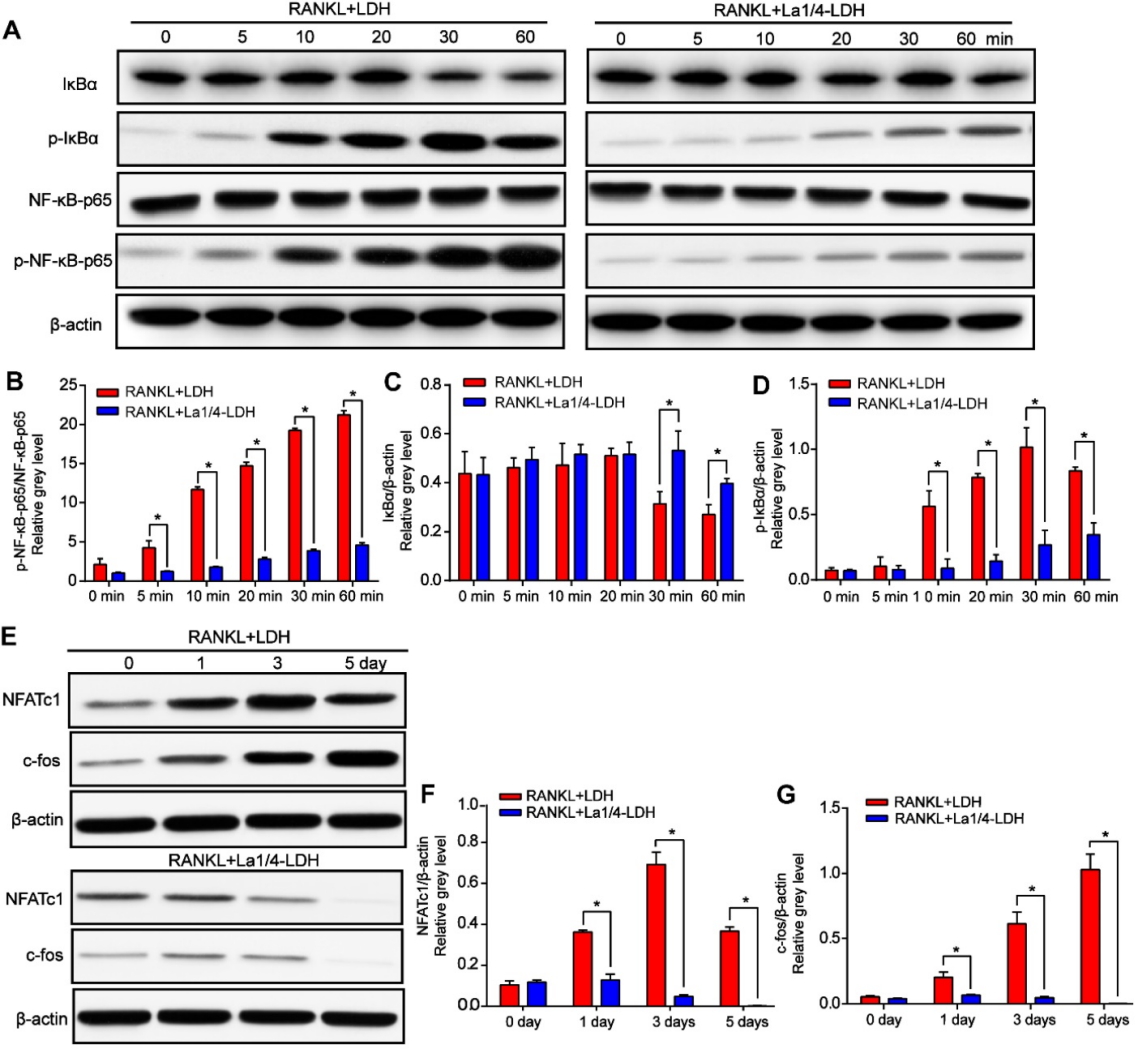
**La-LDH nanohybrid scaffolds inhibited RANKL-mediated osteoclastogenesis.** (A) Western blot analysis of protein expression of NF-κB-p65, p-NF-κB-p65, IκBα and p-IκBα in the BMMs pre-treated with the extracts of La1/4-LDH or LDH scaffolds for 4 h followed by the stimulation with 50 ng/mL RANKL. β-actin acted as an internal control. (B) The levels of p-NF-κB-p65 were normalized to total levels of NF-κB-p65 (*p < 0.05). The levels of (C) total and (D) phosphorylated IκBα were normalized to β-actin (*p < 0.05). (E) Western blot analysis of protein expression of NFATc1 and c-fos in BMMs stimulated with the extracts of La1/4-LDH or LDH scaffolds in the presence of RANKL for 0, 1, 3, and 5 days. β-actin acted as an internal control. The protein levels of (F) NFATc1 and (G) c-fos were normalized to β-actin (*p < 0.05).

The expression levels of p-IκBα gradually increased with time up to 30 min and then decreased after the BMMs were treated with RANKL and extracts of the LDH scaffolds, whereas this effect was remarkably suppressed for the La-LDH group (Figure 8A and D). IκBα was significantly degraded at 30 min in the LDH group, while the tendency was rescued by the La-LDH scaffolds in the range of 30~60 min (Figure 8A and C). Both the phosphorylation and degradation of IκBα that acted as a unique endogenous protein suppressing the activation of NF‐κB-p65, played an indispensable role in NF‐κB-p65 activation and translocation. The expression levels of p-NF‐κB-p65 in the LDH group were significantly higher than those in the La-LDH groups at different time points (Figure 8A and D), indicating that the La^3+^ dopants in the scaffolds exhibited anti-osteoclastogenic activity via regulating NF‐κB signaling way. For the LDH scaffolds, the protein levels of NFATc1 and c-Fos gradually increased and arrived at their peak values after 3 days and 5 days, respectively (Figure 8E-F). On the contrary, the dramatical attenuation of NFATc1 and c-Fos occurred in the La-LDH group, and their expression levels decreased in a time-dependent manner (Figure 8E-G). Therefore, the La-LDH nanohybrid scaffolds possessed anti-osteoclastogenic activity by regulating NF-κB signaling way.

### *In vivo* bone regeneration of La-LDH nanohybrid scaffolds

**Figure 9:**
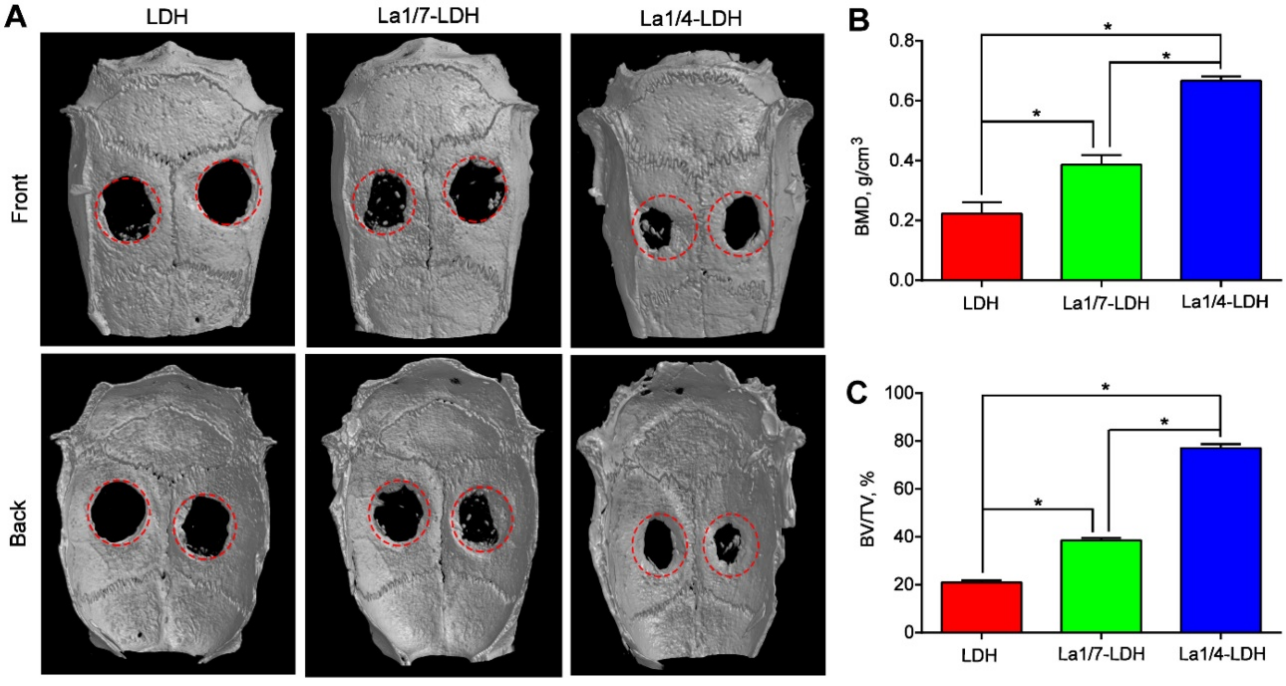
** La-LDH nanohybrid scaffolds promoted *in vivo* bone formation.** (A) Micro-CT images of OVX-rat cranial defects implanted with LDH, La1/7-LDH and La1/4-LDH scaffolds at 12 weeks of post-implantation. (B) BMD and (C) BV/TV ratio for LDH, La1/7-LDH and La1/4-LDH groups (*p < 0.05).

An OVX-rat cranial defect model was created in order to evaluate *in vivo* bone regeneration ability of La-LDH scaffolds (Figure 9). Bone tissues lacked enough self-healing ability to heal critical-sized bone defects especially for osteoporotic patients 44. The representative micro-CT images indicated that very few new bones were detected in the blank control group (Figure S2). Moreover, the OVX-rat cranial defect region remained almost empty in the LDH group even after 12 weeks of implantation, suggesting the poor bone regeneration of LDH scaffolds in osteoporotic conditions. Interestingly, the La1/7-LDH and La1/4-LDH nanohybrid scaffolds significantly triggered the regeneration of osseous tissues, and the defects in the La1/4-LDH group almost filled with new osseous tissues (Figure 9A). To quantitatively analyze the bone regeneration capacity of La-LDH nanohybrid scaffolds, BMD and BV/TV values were calculated according to micro-CT images (Figure 9). For the LDH, La1/4-LDH or La1/7-LDH groups, the BMD values were 0.223 ± 0.038, 0.387 ± 0.032 and 0.667 ± 0.015 g/cm^3^, and the BV/TV values were 20.93 ± 1.06%, 38.57 ± 1.00% and 77.17 ± 1.60%, respectively (Figure 9B and C). The morphometrical analysis results revealed that the La^3+^ dopants in bone scaffolds facilitated new osseous tissue formation, and the bone regeneration capacity was enhanced with the increase of La^3+^ doping amounts.

**Figure 10:**
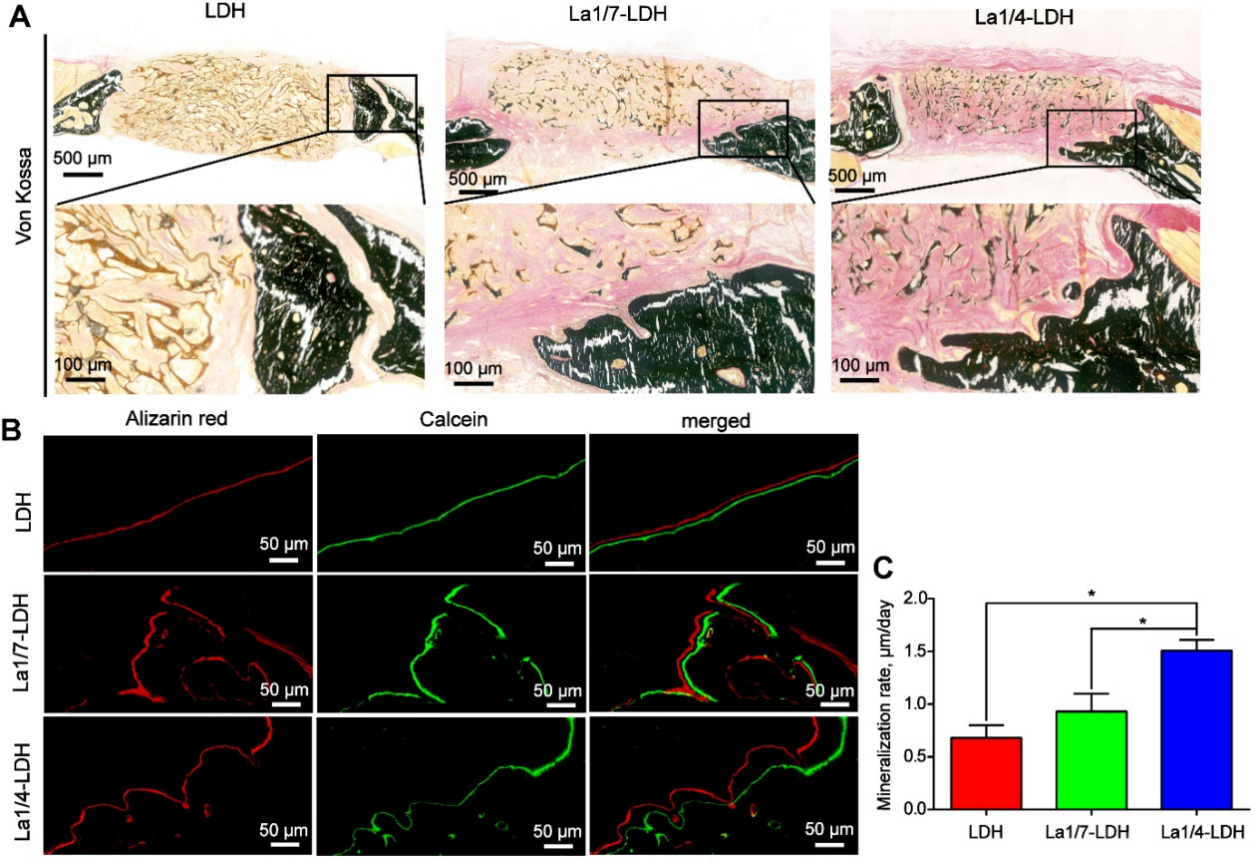
**La-LDH nanohybrid scaffolds accelerated bone mineralization.** (A) Von Kossa staining images of OVX-rat cranial defects implanted with bone scaffolds at 12 weeks of post-implantation. (B) Fluorochrome-labeling analysis of bone mineralization by calcein (green) at 14 days and alizarin red (red) at 7 days before euthanasia. (C) Quantitative analysis of mineralization rates for LDH, La1/7-LDH and La1/4-LDH groups (*p < 0.05).

The Von Kossa stained images revealed that only marginal mineralized tissues at the defect periphery were detected in the LDH group (Figure 10A). Both the La1/7-LDH and La1/4-LDH scaffolds supported the formation of mineralized tissue bridging, and the newly regenerated bone tissues gradually grew into the interior of the bone scaffolds (Figure 10A). In order to investigate osteogenic mineralization rates, double fluorescent labeling with calcein and alizarin red was conducted at 14 days and 7 days before euthanasia, respectively (Figure 10B). For the LDH, La1/7-LDH and La1/4-LDH groups, the mineralization rates were 0.68 ± 0.12, 0.93 ± 0.17 and 1.51 ± 0.1 μm/day, respectively (Figure 10C). The higher mineralization rates in the La-LDH groups than the pure LDH groups indicated that the La^3+^ dopants facilitated the bone formation and mineralization even in an osteoporotic condition.

**Figure 11:**
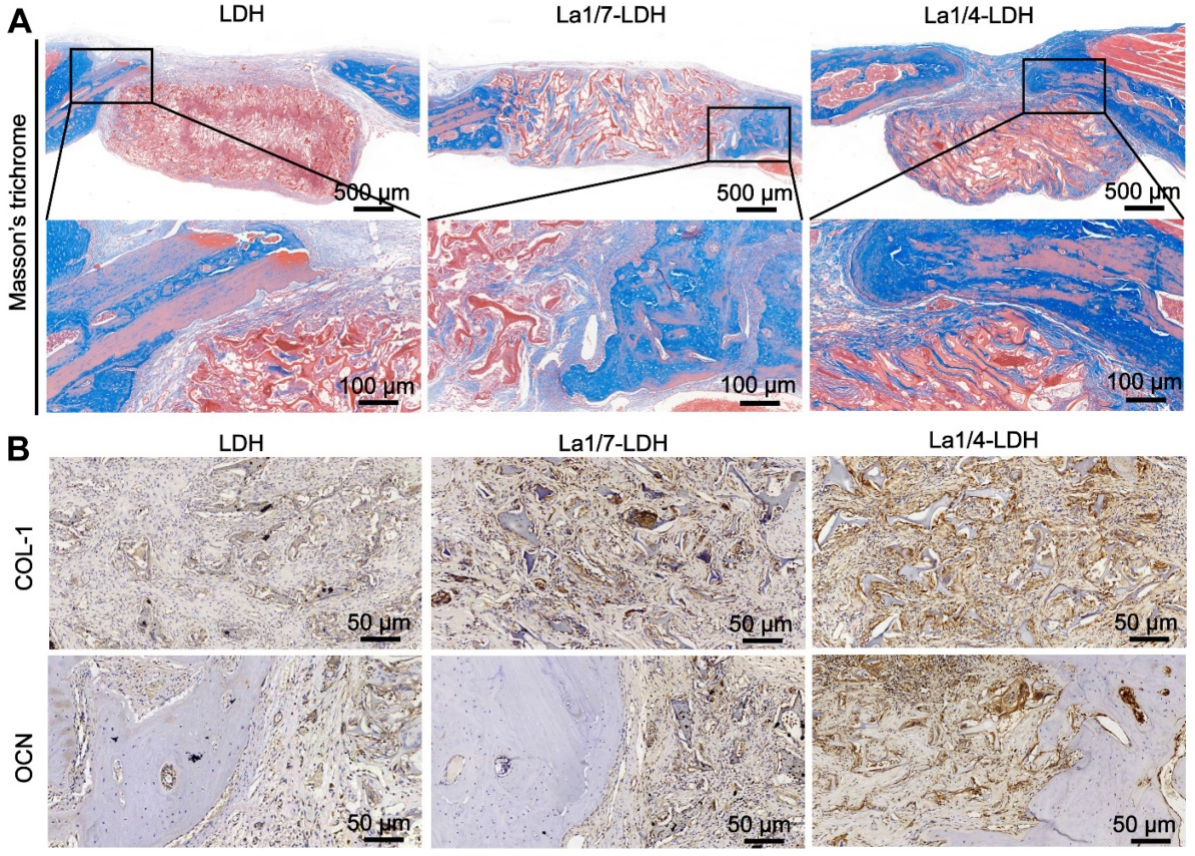
**La-LDH nanohybrid scaffolds facilitated collagen deposition and osteocalcin formation.** (A) Masson's trichrome staining images of collagen components in the LDH, La1/7-LDH and La1/4-LDH groups. (B) Immunohistochemical staining images of COL-I and OCN for LDH, La1/7-LDH and La1/4-LDH groups.

After 12 weeks of post-implantation, the collagen deposition in the decalcified specimens was analyzed by Masson's trichrome staining images (Figure 11). For the LDH group, the bone defect sites were filled with fibrous tissues without distinct collagen deposition. Notably, a large number of collagen components were detected in the defect sites implanted with the La1/7-LDH and La1/4-LDH nanohybrid scaffolds (Figure 11A). The three-dimensional macropores in the La-LDH nanohybrid scaffolds induced the collage formation from the defect margin into the scaffold center. Especially for the La1/4-LDH group, the defective zone nearly disappeared (Figure 11A). The immunohistochemical staining images further proved that La-LDH nanohybrid scaffolds significantly promoted the OCN and COL-1 formation, and the corresponding deposition amounts had positive correlation with the La^3+^ doping amounts in the scaffolds (Figure 11B). Taken together, the results of OVX-rat cranial defect models provided the sufficient evidence that the La^3+^ dopants in the La-LDH scaffolds contributed to new bone tissue regeneration in osteoporotic conditions.

## Discussion

For osteoporotic patients, the balance between bone formation and resorption is struck on account of the attenuated osteogenic differentiation ability of BMSCs and excessive osteoclastogenesis, leading to the difficult therapy of osteoporosis-related bone defects 45,46. Ca is one of main chemical elements in bone minerals, which promotes cellular infiltration, osteogenic differentiation and matrix mineralization 47,48. The synthetic calcium-based scaffolds such as β-tricalcium phosphate and hydroxyapatite can support osteogenesis for bone defect healing, but lack enough activity to retard osteoclastogenesis 49-51. Lanthanum element plays an important role in modulating the balance of osteoblast and osteoclast differentiation 11-13. Hence, it is inferred that the La^3+^ ions in La-LDH nanohybrid scaffolds may simultaneously activate osteogenesis and inhibit osteoclastogenesis for osteoporosis-related bone defect healing.

A freeze-drying strategy was used to construct the La-LDH nanohybrid scaffolds by using La-LDH nanoplates and CS as original materials. The La-LDHs were composed of positively charged metal hydroxide layers and negatively charged interlayer anions with crystal water 21. The hydroxyl groups and crystal water in La-LDHs were easily attach to CS via hydrogen bonding, resulting in the ordered arrangement of two-dimensional La-LDH nanoplates within CS matrix via a self-assembly procedure (Figure 1B). The mechanical property of bone scaffolds was related to their microstructures 40,52. The ordered arrangement structure and organic/inorganic nanohybrid characteristic remarkably increased the compressive strength of the La-LDH nanohybrid scaffolds up to 0.21 MPa (Figure 3B). The appropriate mechanical property of the La-LDH scaffolds could effectively prevent the damages of the interconnected macropores, supporting the in-growth of bone tissues (Figures 10 and 11).

The La-LDH nanohybrid scaffolds possessed the good cytocompatibility and histocompatibility (Figures 4-11), which depended mainly on their porous structure and chemical component. The 3D interconnected macropores with pore sizes of 100~200 μm not only facilitated the adhesion and pseudopodium migration of rBMSCs-OVX along the pore walls (Figure 4A), but also promoted the in-growth of the newly formed bone tissues from the surfaces into the interiors (Figures 10 and 11). The biological effects of REEs presented the hormetic concentration-dependent tendency with positive effects at low concentrations whereas adverse effects at high concentrations 53. Even after the La1/4-LDH nanohybrid scaffolds were immersed* in vitro* for 5 days, the La^3+^ concentrations of ~0.35 μM were kept in the safe dosage range (Figure 3C). The La^3+^ ions in the La-LDH scaffolds significantly accelerated the proliferation of rBMSCs-OVX, and had none apparent toxicity to BMMs (Figure 4B and C). In addition, no pathological change was detected in the H&E staining images of heart, liver, spleen, lung and kidney after 12 weeks of implantation, suggesting that the La^3+^ ions derived from the *in vivo* biodegradation of La-LDH scaffolds had non-toxicity to other normal tissues (Figure S3).

The weakened osteogenic differentiation potential of BMSCs derived from osteoporotic patients was partially ascribed to the inordinate bone turnover in osteoporotic condition 54,55. Fortunately, the La^3+^ dopants in the La-LDH scaffolds significantly enhanced the ALP activity and calcium nodule deposition (Figure 5A and B), and up-regulated the expression levels of osteogenic-related genes such as ALP, Runx-2, COL-1 and OCN (Figure 5D-G). The osteogenic differentiation effects of rBMSCs-OVX positively correlated with the doping amounts of La^3+^ ions in the La-LDH scaffolds (Figure 5). As compared with the LDH group, the La-LDH groups up-regulated the expression of p-GSK-3β and β-catenin as well as downstream target protein Runx-2 (Figure 6A-D), suggesting that the La^3+^ dopants augmented the osteoinductivity of the La-LDH nanohybrid scaffolds via activating Wnt/β-catenin pathway 56,57. Under the stimulus of upstream signaling, β-catenin accumulated in the cytoplasm and subsequently translocated to the nucleus, which brought about downstream osteogenic-related genes transcription. The inhibition of GSK-3β was proven to restore the β-catenin level to some extent, and then facilitated osteogenesis differentiation of BMSCs 58,59. The OVX-rat cranial defect model further proved that the La-LDH nanohybrid scaffolds promoted the *in vivo* formation of the collagenous and non-collagenous organic matrix compared to the LDH scaffolds (Figure 11).

To effectively treat osteoporosis-related bone defects, ideal bone scaffolds should possess the anti-osteoclastogenic activity, too 60. The estrogen deficiency in OVX rats caused the increase of RANKL level, which was regarded as a crucial cytokine inducing osteoclast differentiation and survival 61. The La1/7-LDH and La1/4-LDH nanohybrid scaffolds remarkably down-regulated the expression of RANKL due to the presence of La^3+^ dopants (Figure 6A and F). OPG, a competitive receptor of RANKL, blocked the binding interaction between RANKL and RANK, and thus inhibited the activation and differentiation of osteoclasts 62. The La^3+^ dopants in the LDH scaffolds significantly up-regulated expression level of OPG, and augmented the OPG/RANKL ratio of rBMSCs-OVX (Figure 6). The La^3+^ dopants in the La-LDH scaffolds remarkably enhanced the anti-osteoclastogenic activity, as confirmed by the fewer TRAP-positive osteoclasts and lower expression of osteoclast-specific genes than the control group (Figure 7A-J). The suppressive mechanism of the La-LDH scaffolds on RANKL-mediated osteoclastogenesis was related to NF-κB pathway. The activation of NF-κB pathway led subsequently to the induction and activation of NFATc1 and c-Fos, which were deemed as the master transcription factors responsible for initiating osteoclast differentiation 63,64. Interestingly, the La-LDH scaffolds exhibited an inhibitory effect on the activation of NF-κB signaling, involving in conspicuously diminished p-IκBα and p-NF-κB-p65 (Figure 8A-D). NFATc1, a key downstream target of NF-κB pathway, could regulate the expression of multiple genes related to osteoclast formation and function 65. Immediately after NF-κB translocates to the nucleus, it bound to the promoter of NFATc1 to induce auto-amplification under the participation of transcription factor c-fos 66. As we hypothesized, the attenuation of NF-κB activation by the La-LDH nanohybrid scaffolds was accompanied with the reduced levels of NFATc1 and c-fos (Figure 8E-G), and subsequently inhibited the expression of multiple target genes including CTSK, CTR, TRAP, V-ATPase d2 and DC‐STAMP (Figure 7). Panzavolta et al. reported that Sr^2+^ ions also possessed the bi-directional regulation functions with the activation of osteogenesis and the inhibition of osteoclastogenesis 53, 67.

*In vitro* experimental results demonstrated that the La-LDH nanohybrid scaffolds not only promoted the osteogenic differentiation of rBMSCs-OVX via the activation of Wnt/β-catenin signaling pathway, but also suppressed RANKL-induced osteoclastogenesis via the inhibition of NF‐κB signaling pathway. These conclusions were further proved by *in vivo* OVX-rat critical-size calvarial defect model. As compared with the LDH scaffolds, the La-LDH nanohybrid scaffolds significantly promoted the new bone formation and matrix mineralization, and the bone regeneration capacity positively correlated with the La^3+^ doping amounts (Figures 9-11). Taken together, the La-LDH nanohybrid scaffolds could be employed as a promising bone scaffold material for osteoporotic bone regeneration.

## Conclusions

For the effective therapy of osteoporosis-related bone defects, we successfully fabricated La-LDH nanohybrid scaffolds with the ordered arrangement of the La-LDH nanoplates on CS matrix. The macropores with sizes of 100~200 μm facilitated the adhesion and pseudopodium migration of rBMSCs-OVX, and promoted the in-growth of the newly formed bone tissues. The La-LDH nanohybrid scaffolds showed the controlled release performance of La^3+^ ions, whose concentrations were kept in the safety range. Interestingly, the La^3+^ dopants in the La-LDH nanohybrid scaffolds not only supported the osteogenic differentiation of rBMSCs-OVX via the activation of Wnt/β-catenin signaling pathway, but also effectively suppressed RANKL-induced osteoclastogenesis via the inhibition of NF‐κB signaling pathway. The bidirectional regulation abilities of La-LDH scaffolds on osteoblasts and osteoclasts made them promote *in vivo* new bone regeneration in the OVX-rat cranial defects. However, the La-LDH nanohybrid scaffolds lacked the enough mechanical strength to treat bone defects under load-bearing conditions. In the further work, we will focus on the fabrication of high-mechanical strength La-based scaffolds, and investigate the effect mechanism of La^3+^ ions on angiogenesis under osteoporotic conditions.

## Supplementary Material

Supplementary figures and tables.
