# Supplementary material for: Bi-directional regulation functions of lanthanum-substituted layered double hydroxide nanohybrid scaffolds via activating osteogenesis and inhibiting osteoclastogenesis for osteoporotic bone regeneration
Source: Theranostics. 2021 May 3;11(14):6717–34. doi: 10.7150/thno.56607 (PMC8171081; doi:10.7150/thno.56607)
Supplement: Supplementary file 1 — Supplementary figures and tables. [file thnov11p6717s1.pdf]

Supplementary materials

Bi-directional regulation functions of lanthanum-substituted layered double hydroxide nanohybrid scaffolds *via* activating osteogenesis and inhibiting osteoclastogenesis for osteoporotic bone regeneration

Min Chu[a,]† Zhenyu Sun,[b,]† Zhanghao Fan,[a] Degang Yu,[b] Yuanqing Mao[b,*] and Yaping Guo[a,*]

[1] The Education Ministry Key Lab of Resource Chemistry and Shanghai Key Laboratory of Rare Earth Functional Materials, Shanghai Normal University, Shanghai 200234, China

[2] Shanghai Key Laboratory of Orthopedic Implants, Department of Orthopedic Surgery, Shanghai Ninth People's Hospital, Shanghai JiaoTong University School of Medicine, Shanghai 200011, China

*Corresponding author.

E-mail addresses: ypguo@shnu.edu.cn (YP Guo); yuanqingmao@163.com (YQ Mao)

†Min Chu and Zhenyu Sun contributed equally to this work.

Table S1. The mass amounts of chemical reagents for La-LDH nanoplates.

Chemical reagents	LDHs (g)	La1/7-LDHs (g)	La1/4-LDHs (g)
$Mg(NO_3)_2 \cdot 6H_2O$	23.0787	23.0787	23.0787
$Al(NO_3)_3 \cdot 9H_2O$	11.2540	9.8472	9.0032
$La(NO_3)_3 \cdot 6H_2O$	0	1.6237	2.5980

Table S2. Primer sequences for rat BMSCs-OVX.

Gene	Primers (F=forward; R=reverse)
GAPDH	F: 5'-CCTGCACCACCAACTGCTTA-3'<br>R: 5'-GGCCATCCACAGTCTTCTGAG-3'
ALP	F: 5'-CATCATCATGTTCCTGGGAG-3'<br>R: 5'-GACCTGAGCGTTGGTGTTGT-3'
Runx-2	F: 5'-ATCCAGCCACCTTCACTTACACC-3'<br>R: 5'-GGGACCATTGGGAACTGATAGG-3'
Col-1	F: 5'-CTGCCCAGAAGAATATGTATCACC-3'<br>R: 5'-GAAGCAAAGTTTCCTCCAAGACC-3'
OCN	F: 5'-CAGTAAGGTGGTGAATAGACTCCG-3'<br>R: 5'-GGTGCCATAGATGCGCTTG-3'
OPG	F: 5'-GTCCCTTGCCCTGACTACTCT-3'<br>R: 5'-GACATCTTTTGCAAACCGTGT-3'
RANKL	F: 5'-CCCATCGGGTTCCCATAAAGTC-3'<br>R: 5'-GCCTGAAGCAAATGTTGGCGTA-3'

Table S3. Primer sequences for mouse osteoclasts.

Gene	Primers (F=forward; R=reverse)
GAPDH	F: 5'-GGTGAAGGTCGGTGTGAACG-3'<br>R: 5'-CTCGCTCCTGGAAGATGGTG-3'
NFATc1	F: 5′-TGCTCCTCCTCCTGCTGCTC-3′<br>R: 5′-GCAGAAGGTGGAGGTGCAGC-3′
CTSK	F: 5′-GGGAGAAAAACCTGAAGC-3'<br>R: 5′-ATTCTGGGGACTCAGAGC-3'
V-ATPase d2	F: 5′-AAGCCTTTGTTTGACGCTGT-3′<br>R: 5′-TTCGATGCCTCTGTGAGATG-3’
TRAP	F: 5′-CTGGAGTGCACGATGCCAGCGACA-3’<br>R: 5′- TCCGTGCTCGGCGATGGACCAGA-3’
DC-STAMP	F: 5′-AAAACCCTTGGGCTGTTCTT-3’<br>R: 5′-AATCATGGACGACTCCTTGG-3’
c-Fos	F: 5′-CCAGTCAAGAGCATCAGCAA-3′<br>R: 5′-AAGTAGTGCAGCCCGGAGTA-3′
CTR	F: 5′-TGCAGACAACTCTTGGTTGG-3′<br>R: 5′-TCGGTTTCTTCTCCTCTGGA-3′

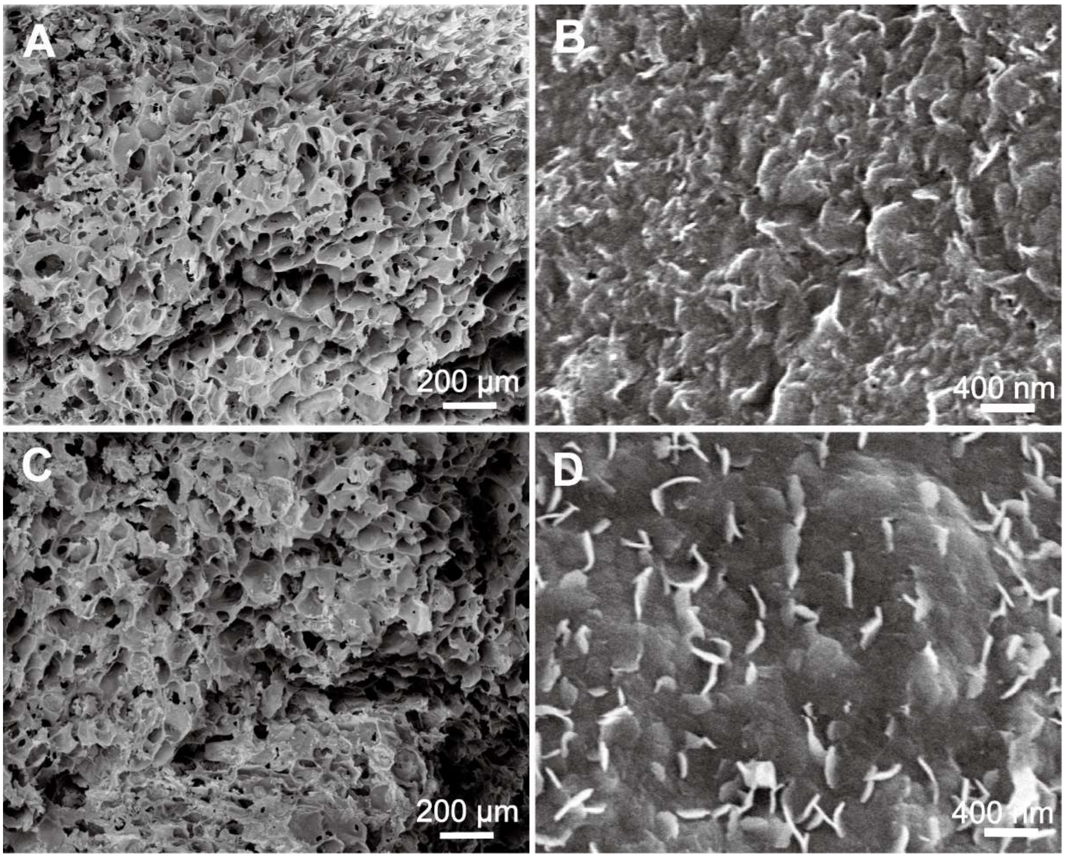

Figure S1. SEM images of samples: (A, B) LDH scaffolds; (C, D) La1/7-LDH scaffolds.

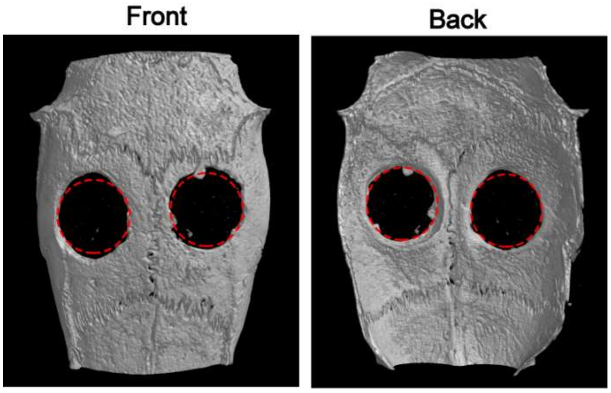

Figure S2. Micro-CT images of OVX-rat cranial defects without any scaffold materials at 12 weeks of post-implantation.

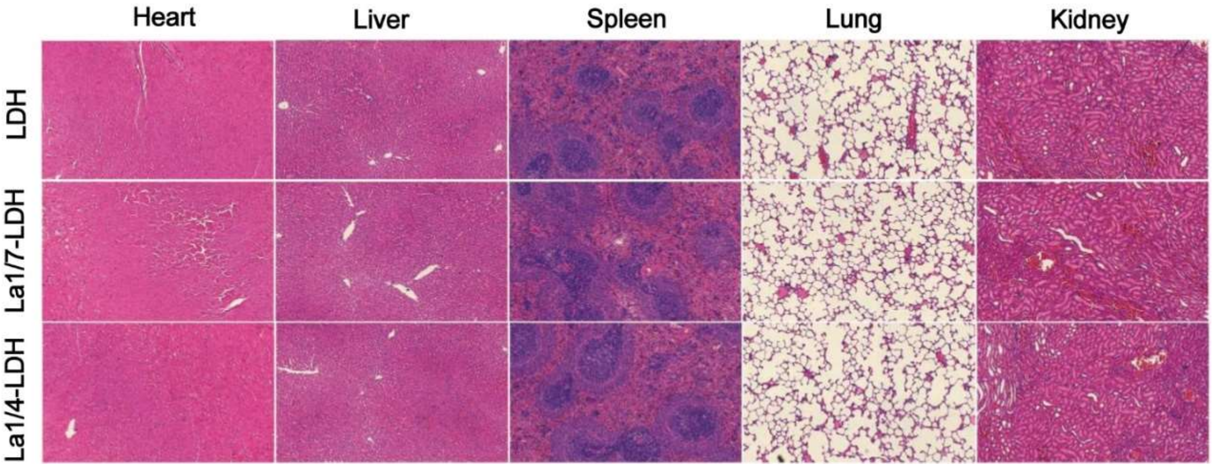

Figure S3. H&E staining images of rat heart, liver, spleen, lung and kidney after 12 weeks of implantation
